# Qigong Exercise Alleviates Fatigue, Anxiety, and Depressive Symptoms, Improves Sleep Quality, and Shortens Sleep Latency in Persons with Chronic Fatigue Syndrome-Like Illness

**DOI:** 10.1155/2014/106048

**Published:** 2014-12-25

**Authors:** Jessie S. M. Chan, Rainbow T. H. Ho, Ka-fai Chung, Chong-wen Wang, Tzy-jyun Yao, Siu-man Ng, Cecilia L. W. Chan

**Affiliations:** ^1^Centre on Behavioral Health, The University of Hong Kong, 2/F, Hong Kong Jockey Club Building for Interdisciplinary Research, 5 Sassoon Road, Pokfulam, Hong Kong; ^2^Department of Social Work and Social Administration, The University of Hong Kong, Hong Kong; ^3^Department of Psychiatry, The University of Hong Kong, Hong Kong; ^4^Department of Biostatistics, Harvard School of Public Health, Cambridge, MA, USA

## Abstract

*Objectives*. To evaluate the effectiveness of Baduanjin Qigong exercise on sleep, fatigue, anxiety, and depressive symptoms in chronic fatigue syndrome- (CFS-) like illness and to determine the dose-response relationship. *Methods*. One hundred fifty participants with CFS-like illness (mean age = 39.0, SD = 7.9) were randomly assigned to Qigong and waitlist. Sixteen 1.5-hour Qigong lessons were arranged over 9 consecutive weeks. Pittsburgh Sleep Quality Index (PSQI), Chalder Fatigue Scale (ChFS), and Hospital Anxiety and Depression Scale (HADS) were assessed at baseline, immediate posttreatment, and 3-month posttreatment. The amount of Qigong self-practice was assessed by self-report. *Results*. Repeated measures analyses of covariance showed a marginally nonsignificant (*P* = 0.064) group by time interaction in the PSQI total score, but it was significant for the “subjective sleep quality” and “sleep latency” items, favoring Qigong exercise. Improvement in “subjective sleep quality” was maintained at 3-month posttreatment. Significant group by time interaction was also detected for the ChFS and HADS anxiety and depression scores. The number of Qigong lessons attended and the amount of Qigong self-practice were significantly associated with sleep, fatigue, anxiety, and depressive symptom improvement. *Conclusion*. Baduanjin Qigong was an efficacious and acceptable treatment for sleep disturbance in CFS-like illness. This trial is registered with Hong Kong Clinical Trial Register: HKCTR-1380.

## 1. Introduction

CFS is a complex, medically unexplained, and debilitating condition, which is characterized by persistent fatigue of at least 6 months. Estimated prevalence of CFS ranged between 0.007% and 2.8% of the general adult population [1]. Sleep disturbance presenting as unrefreshing or nonrestorative sleep is one of the diagnostic criteria of CFS and is very common in the patients with CFS [2]. Up to 87–95% of patients with CFS have nonrestorative sleep and the associated daytime dysfunction [3]. Subjective sleep quality was significantly worse in CFS patients compared with healthy controls [4]. Psychiatric comorbidity is also common in chronic fatigue and CFS; over 80% of patients with chronic fatigue and CFS had a lifetime history of psychiatric disorders such as depression or generalized anxiety disorder [5, 6]. People with CFS are likely undertreated for their psychiatric illnesses [7]. A large part of the patients with CFS remains unrecognized by general practitioners in the community [8]. CFS-like illness is defined based on self-reported fatigue characteristics, associated symptoms, and medical history using the similar criteria for CFS but without confirmation by medical examination [9–11]. Thus, CFS-like illness may include chronic fatigue and CFS.

To date, no curative treatment is available for CFS [12] and the treatment is often symptom based [1]. Only cognitive behavior therapy and graded exercise therapy have been shown to be effective in treating fatigue and the associated symptoms of CFS [1, 12]. Pharmacotherapy is commonly used for sleep disturbance and psychiatric symptoms in CFS [13]; however, psychotropic medications, especially hypnotics, have limited clinical benefits and significant side effects and are not recommended for long-term use [14]. Various nonpharmacological therapies have been proposed, and the use of complementary and alternative medicine (CAM) is growing for the treatment of CFS [15]. Various CAM modalities including mind-body intervention [16], exercise therapy [17, 18], and Qigong exercise [15, 19] have demonstrated positive effects in relieving insomnia, chronic fatigue, anxiety, and depressive symptoms. Qigong is an ancient Chinese self-healing mind-body exercise and it contains meditation, breathing, body posture, and gentle movement. According to the traditional Chinese medicine theory, Qigong aims to promote the circulation of vital energy “Qi” in the meridian system (Qi vital energy channel) and to improve the balance of Qi through the regulation of body, mind, and breathing [20].

Our prior study has demonstrated that Qigong exercise not only reduces fatigue and depressive symptoms, but also improves mental functioning and increases telomerase activity in patients with CFS-like illness [21, 22]; however, the effects on sleep remain unclear. A number of studies have reported that aerobic exercise has positive effect on sleep quality in patients with chronic insomnia [23, 24] and obstructive sleep apnea [25]; and several studies have shown that Qigong exercise improves sleep quality in patients with fibromyalgia [26, 27], perimenopausal women [28], and community-dwelling older adults [29]. However, to our knowledge, no study has examined whether Qigong exercise can improve sleep quality in patients with CFS-like illness or assessed the dose-response relationship between Qigong exercise and symptom improvement. In addition, recent systematic reviews on CAM therapies for CFS, insomnia, depression, and anxiety have highlighted that limited scientific evidence is available and more rigorous randomized controlled trials (RCTs) with adequate sample size and appropriate controls are warranted [15, 19, 30]. Thus, the primary objective of this large-scale RCT was to evaluate the effectiveness of Baduanjin Qigong exercise on sleep, fatigue, anxiety, and depressive symptoms in patients with CFS-like illness. Baduanjin (also called Eight-Section Brocades, 八段錦) is one of the most common forms of Chinese health Qigong exercise, characterized by its simple, slow, and relaxing movements. The exercise involves 8 simple movements, according to the traditional Chinese medicine theory, each of which can enhance the function of certain organs or parts of the body [31]. As Baduanjin Qigong is very easy to learn and less physically or cognitively demanding, it is popular in the Chinese population as a safe Qigong exercise to promote health [31]. The secondary objective was to investigate the dose-response relationship between Qigong exercise and symptom improvement.

## 2. Methods

### 2.1. Study Design

This was a randomized, waitlist-controlled, parallel-group study. Major assessments were at baseline (T0), immediate postintervention (T1), and 3-month postintervention (T2). Participants were randomly assigned to either Qigong exercise or waitlist. Randomization was done using computer-generated random numbers. Blinding of the participants was not possible due to the nature of intervention. Sample size was calculated based on changes in Chalder Fatigue Scale (ChFS) score. According to our prior study [21], we expected a between-group difference of 6.5 points on the ChFS, equivalent to an effect size of 0.64. A sample size of 51 in each group would have a power of 80% to detect the 6.5-point between-group difference in total ChFS score at a significance level of 0.05. Allowing a 30% dropout, we estimated that this study would require a sample size of 75 in each group. Ethics approval was obtained from the local institutional review board. The study was registered in the Hong Kong Clinical Trial Register (number HKCTR-1380). We followed the CONSORT recommendations in designing and reported the controlled trial [32].

### 2.2. Participants

After the study was advertised in the media, a total of 3848 Chinese adults from the local community were interested in the study and completed an online screening questionnaire. The questionnaire was set according to the US Centers for Disease Control and Prevention (CDC) diagnostic criteria for CFS [33]. Participants were diagnosed as having CFS-like illness if they had unexplained, persistent fatigue of new onset (not lifelong) for 6 or more months, accompanied by 4 or more of the following 8 symptoms: unrefreshing sleep, new headaches, impaired memory or concentration, postexertional malaise, muscle pain, multijoint pain, sore throat, and tender lymph nodes, and did not report any history of cancer, hypothyroidism, sleep apnea, narcolepsy, hepatitis B or C virus infection, severe obesity, and mental disorders, including major depressive disorder, schizophrenia, and bipolar disorder, and alcohol or other substances abuse based on a medical history checklist [33]. To minimize the impact of other undiagnosed chronic illnesses, we excluded people older than 50 years who were more likely to have chronic illnesses presenting with chronic fatigue than younger participants. We also excluded people who had participated in Qigong practice in the past 6 months.

**Figure 1:**
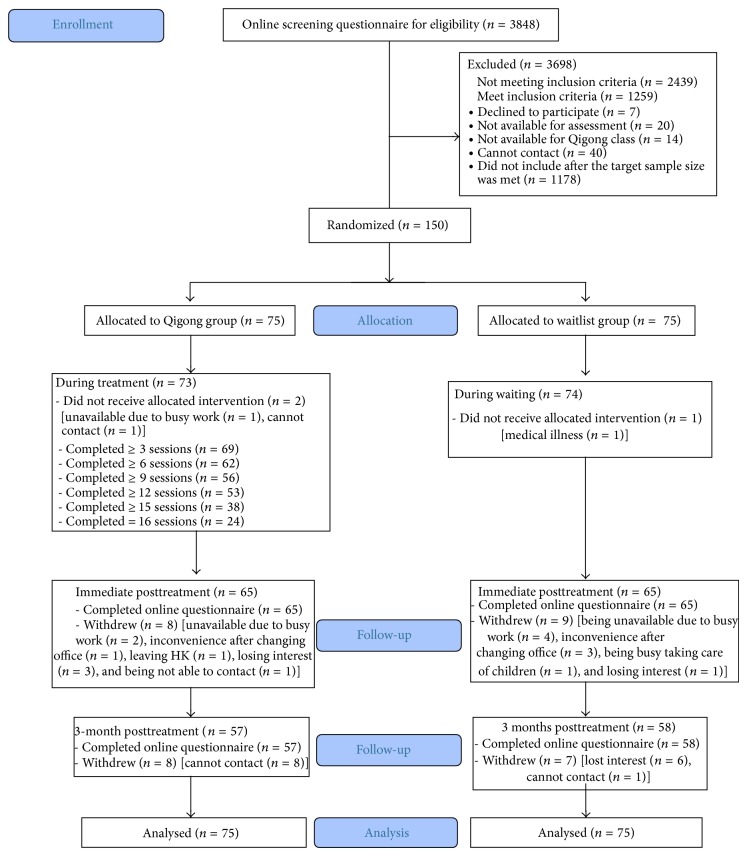
Recruitment flowchart.

The participants were firstly screened through online questionnaires. If there was any uncertainty concerning eligibility, the online questionnaires were reviewed by 2 investigators and any discrepancies were resolved by discussion. A computer-generated list of random numbers was used for allocation of the participants. We randomly selected from among 1409 eligible subjects and contacted them by phone until the target sample size was met. The randomization procedure was done prior to phone contact with the potential subjects.  Figure 1 presents the participant flowchart.

### 2.3. Procedure

All participants gave written informed consent prior to further assessment and intervention. At T0, T1, and T2, participants completed a set of questionnaires on sleep, fatigue, anxiety, and depression. Participants were advised to sign an attendance record prior to each class. Daily self-practice of Qigong and other exercises and any adverse events were recorded on a standard log sheet, which were collected weekly during the intervention period and monthly after intervention. However, a majority of the participants did not return their log sheet after intervention; hence, we only analyzed the Qigong self-practice data during the intervention period. No subjects were paid any monetary rewards for participation. However, the Qigong class was provided free of charge and those who completed all assessments and had an attendance rate ≥ 80% would be given a brief health report based on their own data and a DVD on Baduanjin Qigong exercise.

### 2.4. Intervention

Baduanjin Qigong, comprising 8 standardized movements, has been endorsed by the Sports and Culture Commission of the People's Republic of China (PRC). Sixteen sessions of Baduanjin Qigong group training were provided over nine consecutive weeks. Each session lasted 1.5 hours and was conducted by an experienced Qigong master with more than 20 years of experience in teaching and 7-8 assistant Qigong teachers who had 3–5 years of experience in Qigong practice and were certified in teaching Qigong. The session began with body stretching and relaxation (15 minutes), followed by introduction and demonstration of each movement, explanation of the precautions in Qigong exercise, and answering of the questions raised by the participants (25 minutes), then a group Qigong training involving 75 participants (20 minutes) and lastly a small group Qigong practice involving 15 participants with individual guidance by the assistant Qigong teacher who helped to correct the movement of the participants, and the experienced Qigong master oversaw all small groups and provided additional advice (30 min). Participants in the intervention group were advised to practice Qigong for at least 30 minutes every day. Participants in the waitlist group were advised to keep their lifestyle as usual and refrain from joining any Qigong class.

### 2.5. Measures

#### 2.5.1. Screening Measures and Demographics

The online screening questionnaire includes (1) the CDC diagnostic checklist for CFS [33]; (2) a list of medical illnesses based on the CDC exclusion criteria for CFS; (3) sociodemographics, including age, gender, employment status, educational level, marital status, number of children, religious affiliation, and monthly income; (4) lifestyle variables, including exercise habit, smoking, and alcohol drinking; and (5) weight and height.

#### 2.5.2. Pittsburgh Sleep Quality Index (PSQI)

The PSQI is a widely used 19-item self-administered instrument to assess sleep quality and disturbances over a 1-month period [34]. It contains 7 components, subjective sleep quality, sleep latency, sleep duration, sleep efficiency, sleep disturbance, use of sleep medication, and daytime dysfunction. The total score ranges from 0 to 21 and a higher score indicates poorer sleep quality. The Chinese version of PSQI has been shown to have adequate psychometric properties [35, 36].

#### 2.5.3. Chalder Fatigue Scale (ChFS)

The ChFS is a 14-item self-rating scale on the severity of physical fatigue (8 items) and mental fatigue symptoms (6 items) [37]. A higher score is suggestive of greater severity of fatigue. The Chinese version of the ChFS has been shown to be valid and reliable in Chinese adults [38].

#### 2.5.4. Hospital Anxiety and Depression Scale (HADS)

The HADS is a 14-item self-assessment scale on anxiety (7 items) and depressive symptoms (7 items) [39]. Each item can be scored on a 0–3 scale and a higher score denotes a higher level of anxiety or depressive symptoms. The Chinese version of the HADS has satisfactory psychometric properties [40].

#### 2.5.5. Global Assessment, Satisfaction, and Adverse Events

Participants were asked to compare their condition before and after joining the Qigong lessons as very marked improvement, marked improvement, same as before, marked deterioration, and very marked deterioration and whether they were satisfied with the lessons, from very satisfied to very dissatisfied. Participants reported any adverse events by free text in the log sheet.

### 2.6. Data Analysis

All analyses were conducted with Statistical Package for the Social Sciences (SPSS version 18.0, SPSS Inc., Chicago, IL). Continuous data were presented by mean and standard deviation (SD). Categorical data were presented by frequency (percentage). Between-group differences at baseline were assessed by chi-squared test for categorical data and independent *t*-test for continuous data. Repeated measures analyses of covariance (ANCOVA) were performed to assess the interaction effect of group and time for each outcome measure, adjusting for gender, due to a significant between-group difference in gender ratio. The within-group changes were assessed by paired *t*-test and the between-group differences were evaluated by independent *t*-test. Effect size was calculated by Cohen's *d* statistics. The data analysis was conducted based on intention-to-treat principle. The missing values were substituted by the last observed values. The dose-response relationship was assessed using correlation analysis between symptom change scores and the number of Qigong classes attended and the average self-reported Qigong practice per week in minutes.

## 3. Results

### 3.1. Baseline Characteristics of the Participants

Table 1 describes the sociodemographic and lifestyle variables. The mean age was 39.1 years (SD = 7.8) in the Qigong group and 38.9 years (SD = 8.1) in the waitlist group. The participants were predominantly female, employed full time, highly educated, and married. The most often reported duration of fatigue was 1-2 years (*n* = 61, 40.7%), followed by 2–5 years (*n* = 56, 37.3%) and more than 5 years (*n* = 33, 22.0%). The most frequent associated complaint was sleep problems (*n* = 144, 96.0%), followed by muscle pain (*n* = 143, 95.3%), impaired memory or concentration (*n* = 132, 88.0%), postexertional malaise (*n* = 106, 70.7%), arthralgia (*n* = 84, 56.0%), headache (*n* = 82, 54.7%), tender cervical or axillary lymph nodes (*n* = 62, 41.3%), and recurrent sore throat (*n* = 55, 36.7%). A majority of the participants had neither seen any doctors for their fatigue problem (*n* = 117, 78.0%) nor used any medications as treatment (*n* = 138, 92.0%). Except for gender distribution, there were no statistically significant differences in sociodemographic and lifestyle variables between the two groups.

Two participants in the Qigong group dropped out prior to intervention and 8 participants dropped out at immediate posttreatment; in the waitlist group, there was 1 participant who dropped out prior to intervention and 9 dropped out at immediate posttreatment (chi-square test, *P* > 0.05). None of the participants in the Qigong group dropped out due to adverse event (Figure 1).

As shown in Table 2, at baseline, the mean PSQI score was 10.0 and 10.2, while the mean ChFS total score was 37.4 for the Qigong group and 36.4 for the waitlist group, respectively. All clinical measures were balanced between the two groups at baseline.

### 3.2. Efficacy

#### 3.2.1. Sleep

The PSQI total score in the Qigong group was significantly lower at T1 and T2, compared to T0 (*d* = −0.51 and −0.48, resp., all *P* values < 0.001) (Table 2). In the waitlist group, the PSQI total score was also significantly improved at T1 and T2, compared to T0 (*d* = −0.19 and −0.25, *P* = 0.024 and 0.042, resp.). The improvement in the PSQI total score in the Qigong group was significantly greater than that in the waitlist group at T1 (*P* = 0.019) but not at T2 (*P* = 0.149). The group by time interaction in the PSQI total score was marginally nonsignificant (*F*(2,284) = 2.777, *P* = 0.064).

However, there was significant group by time interaction in the “subjective sleep quality” (*F*(2,288) = 6.364, *P* = 0.002) and “sleep latency” (*F*(2,294) = 3.168, *P* = 0.044) items of the PSQI, while the “sleep disturbance” item was marginally nonsignificant (*F*(2,294) = 2.802, *P* = 0.062). The “subjective sleep quality” in the Qigong group was significantly improved at T1 and T2 compared with the waitlist group (*P* = 0.003 and 0.004, resp.). The improvement in “sleep latency” in the Qigong group was only significant at T1 (*P* = 0.019) but not at T2 (*P* = 0.264), compared with the waitlist group (Table 2).

#### 3.2.2. Fatigue

As shown in Table 2, compared with baseline values, the ChFS total score (*d* = −1.19), ChFS physical score (*d* = −1.19), and ChFS mental score (*d* = −0.92) were significantly improved in the Qigong group immediately after intervention (T1) (all *P* values < 0.001), and the improvements were maintained at T2 (all *P* values < 0.001). In the waitlist group, the total fatigue score (*d* = −0.45, *P* < 0.001), physical fatigue score (*d* = −0.44, *P* < 0.001), and mental fatigue score (*d* = −0.35, *P* < 0.001) were also significantly improved at T1 and T2 (all *P* values < 0.001), but the magnitudes of improvement were much smaller. Compared with the waitlist group, the Qigong group had significantly greater improvements in ChFS fatigue score (*F*(2,294) = 16.650, *P* < 0.001), ChFS physical score (*F*(2,294) = 18.527, *P* < 0.001), and ChFS mental score (*F*(2,294) = 9.290, *P* < 0.001).

#### 3.2.3. Anxiety and Depression

As shown in Table 2, there are significant group by time interactions in the HADS anxiety and depression scores. The reduction in anxiety and depressive symptoms was significantly greater in the Qigong group, compared to the waitlist group (HADS anxiety: *F*(2,294) = 4.172, *P* = 0.016; HDAS depression: *F*(2,294) = 10.262, *P* < 0.001).

### 3.3. Dose-Response Relationship

Two participants never came to Qigong lessons. Sixty-two of the 75 participants (82.7%) attended at least 6 of the 16 lessons and 56 participants (74.7%) attended at least 9 lessons (Figure 1). The average number of Qigong sessions attended was 11.9 (SD = 5.1, range = 0–16). There was significant relationship between the number of Qigong sessions attended and the reduction in PSQI, ChFS, and HADS scores from T0 to T1 and from T0 to T2 (Table 3).

Only 64 of the 75 participants returned their log sheet on Qigong self-practice for at least 2 weeks and their self-practice data were included in the analysis. The mean duration of Qigong self-practice was 145.4 minutes per week (SD = 77.2). The amount of Qigong self-practice was significantly associated with the ChFS and HADS change scores from T0 to T1 and from T0 to T2. However, only the “subjective sleep quality” and “sleep disturbance” items of the PSQI from T0 to T1 and the “subjective sleep quality” item from T0 to T2 had significant dose-response relationships with the amount of Qigong self-practice during the intervention period. The dose-response relationship between symptom improvement and the amount of Qigong self-practice remained unchanged after controlling the amount of time spent on other exercises (Table 3).

### 3.4. Global Assessment, Satisfaction, and Adverse Events

Only 53 of the 75 participants in the intervention group returned their questionnaire on global assessment and satisfaction. Thirty-six of the 53 participants (67.9%) felt they had marked or very marked improvement after the Qigong lessons, while the others reported being the same as before. Forty-seven of the 53 participants (88.7%) were satisfied or very satisfied and the rest was neutral. The most common adverse event was muscle ache (*n* = 24), followed by palpitation (*n* = 4), giddiness (*n* = 3), knee pain (*n* = 2), backache (*n* = 2), fatigue (*n* = 2), nervousness (*n* = 2), Qi movement inside body (*n* = 2), dizziness (*n* = 2), shoulder pain (*n* = 1), chest tightness (*n* = 1), shortness of breath (*n* = 1), and increased dreaming (*n* = 1).

## 4. Discussion

To the best of our knowledge, this is the first large scale RCT to investigate the effect of Qigong exercise on sleep quality in persons with CFS-like illness and the dose-response relationship of Qigong. The results indicated that Baduanjin Qigong exercise was more efficacious than waitlist control in relieving sleep, fatigue, anxiety, and depressive symptoms in CFS-like illness. Although the group by time interaction was marginally nonsignificant (*P* = 0.062) for PSQI total score, it was significant for the “subjective sleep quality” and “sleep latency” items of the PSQI. The significant improvement in “subjective sleep quality” was maintained at 3-month postintervention, suggesting that Baduanjin Qigong exercise had at least short-term effect. Most of the participants were satisfied with Qigong exercise and felt they had greatly benefited from the intervention. Three-quarters of the participants attended more than half of the Qigong lessons arranged. Except muscle ache, adverse events were uncommon. Our findings are in line with the growing evidence on the benefits of Qigong for CFS-like illness [21, 22]. In light of the efficacy, acceptability, low cost, and accessibility of Qigong exercise, it should be tested in future studies as an entry-level treatment in a stepped care model for insomnia [41].

Compared with waitlist group, participants receiving Baduanjin Qigong exercise had better sleep quality, shorter sleep latency, and longer sleep duration at immediate postintervention; however, only the improvement in sleep quality was maintained at 3-month postintervention. It was likely that after the lessons participants had not continued to practice Qigong themselves; hence, the early improvement would not be maintained. However, due to the missing data on Qigong self-practice after the intervention period, future studies are needed to confirm our hypothesis. The beneficial effects of Baduanjin Qigong exercise on sleep quality have been shown in previous studies of smaller sample size and on perimenopausal women [28], patients with fibromyalgia [26, 27], and community-dwelling older adults [29]. Our results suggested that Qigong exercise was efficacious in the treatment of sleep disturbance in CFS-like illness. In line with previous studies [27, 28], there was a direct relationship between symptom improvement and the amount of Qigong practice.

We showed that Baduanjin Qigong exercise significantly reduced fatigue, anxiety, and depressive symptoms in patients with CFS-like illness, replicating part of the results in our previous studies that examined another Qigong exercise, named Wu Xing Ping Heng Gong. In our previous studies, Wu Xing Ping Heng Gong improved fatigue and depressive symptoms [21, 22] but not anxiety symptoms. The longer duration of intervention probably accounted for the effectiveness of Qigong for anxiety in this study. There were only 10 sessions of Qigong exercise in 5 weeks in our previous studies instead of 16 sessions in 9 weeks in this study. Another possible reason was that in this study we strongly advised the participants to have daily Qigong self-practice for at least 30 minutes and to keep a personal log sheet. The advice on self-practice and self-monitoring might have encouraged the self-practice of Qigong and enhanced its effectiveness. Our finding of the positive effect of Qigong exercise on anxiety was in line with the conclusion of a recent systematic review [42].

Although the findings of our study were promising, there were several notable limitations. First, our participants were recruited from the community solely based on self-report and they did not receive a thorough physical and mental state examination; hence, it was possible that some of them may not fully meet the CDC criteria for CFS. However, through online questionnaires and exclusion of people older than 50 years, the chance that the chronic fatigue is caused by medical and psychiatric conditions was reduced. Due to the unknown etiology and lack of effective treatment for CFS, a large proportion of CFS patients remains unrecognized and undertreated in the community [7, 8]; it is very difficult to recruit participants with diagnosed CFS. Another limitation was that participants were not blind to the intervention and therefore might have high expectations in the effectiveness of treatment, which could inflate the response; however, improvements were maintained at 3-month postintervention, suggesting that the beneficial effects of Qigong cannot be due to expectancy alone. Our sample was limited to adults younger than 50 years; hence, the results could not be generalized to the older population. We have not assessed the daytime functioning or used more sophisticated methods, such as sleep diary, actigraphy, and polysomnography, in the assessment of sleep disturbance. Our sample size was estimated based on fatigue scale, and hence the sample size might not be sufficient to detect between-group difference in sleep variables. The amount of Qigong self-practice was assessed by self-report and there were missing data; hence, the results should be treated as preliminary. There still existed other nonspecific factors, such as stretch exercise, didactic teaching from Qigong masters, personal attention, and social support among participants, which may contribute to the improved outcomes. Future studies using a control intervention with all the nonspecific factors included can help to understand the specific therapeutic components of Qigong exercise. Despite these limitations, the present study was the first large-scale RCT that showed the beneficial effects of Qigong exercise on sleep disturbance in CFS-like illness.

In conclusion, we found that 16 sessions of Baduanjin Qigong exercise were an efficacious and acceptable treatment for sleep disturbance, fatigue, anxiety, and depressive symptoms in people with CFS-like illness. Future studies should examine the effectiveness of Qigong exercise in a mixed group of insomnia and insomnia in other comorbid conditions. Strategies to enhance participation in Qigong lesson and regular practice are needed.

## Figures and Tables

**Table 1:** Demographic characteristics and lifestyles variables (*n* = 150).

Variables	Qigong (*n* = 75)		Waitlist (*n* = 75)		*P* value
	Mean (SD)	*N* (%)	Mean (SD)	*N* (%)	
Age (years)	39.1 (7.8)		38.9 (8.1)		0.853
Gender					
Female		46 (61.3%)		62 (82.7%)	0.004
Employment					
Full time		67 (89.3%)		68 (90.7%)	0.401
Part time		1 (1.3%)		3 (4.0%)	
Housewife/unemployed/retired		7 (9.3%)		4 (5.3%)	
Education					
Secondary or below		29 (38.7%)		27 (36.0%)	0.234
Tertiary or undergraduate		36 (48.0%)		30 (40.0%)	
Master or above		10 (13.3%)		18 (24.0%)	
Marital status					
Single		29 (38.7%)		26 (34.7%)	0.242
Married/cohabiting		45 (60.0%)		44 (58.7%)	
Divorced/separated		1 (1.3%)		5 (6.7%)	
Number of children					
0		43 (57.3%)		45 (60.0%)	0.707
1		11 (14.7%)		12 (16.0%)	
2		18 (24.0%)		13 (17.3%)	
3 or more		3 (4.0%)		8 (5.3%)	
Has religious affiliation		33 (44.0%)		29 (38.7%)	0.507
Household monthly income (HK$)					
<10,000		10 (13.3%)		11 (14.7%)	0.378
10,000–19,999		22 (29.3%)		22 (29.3%)	
20,000–29,999		19 (25.3%)		10 (13.3%)	
≥30,000		10 (13.3%)		18 (24.0%)	
No income		4 (5.3%)		3 (4.0%)	
Refused to answer		10 (13.3%)		11 (14.7%)	
Lifestyle variables					
Regular exercise		30 (40.0%)		27 (36.0%)	0.614
Daily smoking		3 (4.0%)		3 (4.0%)	1.000
Alcohol drinking ≥2/week		2 (2.7%)		3 (4.0%)	1.000
Body mass index	22.3 (4.9)		21.6 (3.4)		0.360

**Table 2:** Sleep, fatigue, anxiety, and depressive symptoms at baseline, immediate postintervention, and 3-month postintervention.

	Within-group effects					Between-group effects			
	T0	T1^a^	Effect size	T2^a^	Effect size	T1 − T0^b^	T2 − T0^b^	Time × group^c^	
	Mean (SD)	Mean (SD)	Cohen's *d*	Mean (SD)	Cohen's *d*	Mean (SD)	Mean (SD)	*F*	*P* value
PSQI total									
Qigong (*n* = 73)	10.0 (3.7)	8.2 (3.4)^∗∗∗^	−0.51	8.3 (3.4)^∗∗∗^	−0.48	−1.9 (3.4)^∗^	−1.7 (3.4)	2.777	0.064
Waitlist (*n* = 72)	10.2 (3.8)	9.5 (3.7)^∗^	−0.19	9.3 (3.5)^∗^	−0.25	−0.7 (2.6)	−0.9 (3.5)		
Subjective sleep quality									
Qigong (*n* = 74)	1.93 (0.71)	1.42 (0.74)^∗∗∗^	−0.70	1.49 (0.73)^∗∗∗^	−0.61	−0.51 (0.71)^∗∗^	−0.45 (0.7)^∗∗^	6.364	0.002
Waitlist (*n* = 73)	1.86 (0.71)	1.68 (0.69)^∗^	−0.26	1.75 (0.70)	−0.16	−0.18 (0.67)	−0.11 (0.72)		
Sleep latency									
Qigong (*n* = 75)	1.63 (1.06)	1.23 (0.99)^∗∗∗^	−0.39	1.28 (1.02)^∗∗∗^	−0.34	−0.40 (0.75)^∗^	−0.35 (0.81)	3.168	0.044
Waitlist (*n* = 75)	1.69 (0.97)	1.57 (0.92)	−0.13	1.49 (0.95)^∗^	−0.21	−0.12 (0.70)	−0.20 (0.79)		
Sleep duration									
Qigong (*n* = 75)	1.67 (0.91)	1.44 (0.83)^∗^	−0.26	1.40 (0.81)^∗∗^	−0.31	−0.23 (0.80)^∗^	−0.27 (0.72)	1.905	0.151
Waitlist (*n* = 75)	1.75 (0.84)	1.79 (0.89)	−0.05	1.61 (0.85)	−0.17	0.04 (0.81)	−0.13 (0.83)		
Sleep efficiency									
Qigong (*n* = 75)	0.87 (1.16)	0.72 (1.09)	−0.13	0.96 (1.26)	0.07	−0.15 (1.32)	0.09 (1.29)	0.651	0.522
Waitlist (*n* = 75)	1.09 (1.12)	0.79 (1.15)	−0.26	1.05 (1.26)	−0.03	−0.31 (0.97)	−0.04 (1.41)		
Sleep disturbance									
Qigong (*n* = 75)	1.64 (0.69)	1.40 (0.62)^∗∗∗^	−0.37	1.36 (0.63)^∗∗∗^	−0.42	−0.24 (0.52)	−0.28 (0.48)	2.802	0.062
Waitlist (*n* = 75)	1.73 (0.68)	1.63 (0.65)	−0.15	1.63 (0.61)	−0.15	−0.11 (0.61)	−0.11 (0.63)		
Use of sleep medication									
Qigong (*n* = 74)	0.31 (0.78)	0.28 (0.77)	−0.04	0.30 (0.75)	−0.01	−0.03 (0.83)	−0.01 (0.80)	0.220	0.803
Waitlist (*n* = 73)	0.26 (0.60)	0.32 (0.72)	0.09	0.34 (0.79)	0.11	0.05 (0.72)	0.08 (0.89)		
Daytime dysfunction									
Qigong (*n* = 73)	1.90 (0.82)	1.59 (0.94)	−0.35	1.52 (0.96)	−0.43	−0.32 (0.86)	−0.38 (0.92)	1.381	0.253
Waitlist (*n* = 73)	1.77 (0.77)	1.64 (0.75)	−0.17	1.53 (0.73)	−0.32	−0.12 (0.76)	−0.23 (0.83)		
ChFS total									
Qigong (*n* = 75)	37.4 (6.2)	25.6 (12.6)^∗∗∗^	−1.19	25.2 (12.7)^∗∗∗^	−1.22	−11.8 (11.4)^∗∗∗^	−12.2 (11.9)^∗∗∗^	16.650	<0.001
Waitlist (*n* = 75)	36.4 (8.3)	32.3 (9.7)^∗∗∗^	−0.45	31.1 (10.9)^∗∗∗^	−0.55	−4.1 (6.5)	−5.3 (7.8)		
ChFS physical									
Qigong (*n* = 75)	24.2 (4.0)	16.7 (8.0)^∗∗∗^	−1.19	16.2 (8.1)^∗∗∗^	−1.25	−7.5 (7.3)^∗∗∗^	−8.0 (7.5)^∗∗∗^	18.527	<0.001
Waitlist (*n* = 75)	23.0 (5.0)	20.6 (5.9)^∗∗∗^	−0.44	20.0 (6.7)^∗∗∗^	−0.51	−2.4 (4.1)	−3.1 (5.0)		
ChFS mental									
Qigong (*n* = 75)	13.2 (3.6)	9.0 (5.4)^∗∗∗^	−0.92	9.0 (5.3)^∗∗∗^	−0.93	−4.3 (4.9)^∗∗∗^	−4.3 (5.0)^∗∗^	9.290	<0.001
Waitlist (*n* = 75)	13.3 (4.5)	11.7 (4.6)^∗∗∗^	−0.35	11.2 (4.9)^∗∗∗^	−0.45	−1.7 (3.4)	−2.2 (3.7)		
HADS Anxiety									
Qigong (*n* = 75)	10.9 (3.7)	8.5 (4.0)^∗∗∗^	−0.62	8.8 (4.4)^∗∗∗^	−0.52	−2.3 (3.2)^∗∗^	−2.1 (3.7)^∗^	4.172	0.016
Waitlist (*n* = 75)	11.2 (3.6)	10.4 (4.0)^∗^	−0.21	10.2 (4.0)^∗^	−0.26	−0.8 (3.3)	−1.0 (3.3)		
HADS depression									
Qigong (*n* = 75)	9.4 (3.5)	6.6 (3.7)^∗∗∗^	−0.78	7.2 (4.1)^∗∗∗^	−0.58	−2.7 (3.5)^∗∗∗^	−2.1 (3.9)	10.262	<0.001
Waitlist (*n* = 75)	9.5 (3.4)	8.8 (3.9)^∗^	−0.19	8.5 (4.0)^∗∗^	−0.27	−0.7 (2.9)	−1.0 (3.0)		

PSQI: Pittsburgh Sleep Quality Index; ChFS: Chalder Fatigue Scale; HADS: Hospital Anxiety and Depression Scale; SD: standard deviation; T0: baseline; T1: immediate postintervention; T2: 3-month postintervention.

^a^Compared with baseline using paired *t*-test; ^b^compared the change score between groups using independent *t*-test; ^c^repeated measures ANCOVA adjusting for between-group difference in gender ratio.  ^*∗*^ *P* < 0.05,  ^*∗∗*^ *P* < 0.01, and  ^*∗∗∗*^ *P* < 0.001.

**Table 3:** Correlations between Pittsburgh Sleep Quality Index (PSQI), Chalder Fatigue Scale (ChFS), and Hospital Anxiety and Depression Scale (HADS) change scores with number of Qigong sessions attended and weekly duration of Qigong practice.

	Attendance frequency		Self-practice (min./week)			
	(*n* = 75)		(*n* = 64)			
Mean (SD)	11.9 (5.1)		145.4 (77.2)			
Median	15.0		151.7			
Interquartile	(8.0–16.0)		(105.8–185.9)			

T1 − T0	*R*	*P*	*R*	*P*	*R* ^a^	*P* ^a^

Change in PSQI	−0.288	0.013	−0.093	0.474	−0.101	0.439
Change in PSQI-subjective sleep quality	−0.422	0.001	−0.300	0.017	−0.315	0.013
Change in PSQI-sleep latency	−0.321	0.005	−0.205	0.104	−0.189	0.137
Change in PSQI-sleep duration	−0.089	0.445	0.107	0.401	0.115	0.371
Change in PSQI-sleep efficiency	−0.055	0.638	0.122	0.338	0.110	0.389
Change in PSQI-sleep disturbance	−0.266	0.021	−0.318	0.010	−0.303	0.018
Change in PSQI-use of sleep medication	0.039	0.743	0.007	0.956	−0.030	0.816
Change in PSQI-daytime dysfunction	−0.213	0.070	−0.083	0.520	−0.081	0.536
Change in total fatigue	−0.587	<0.001	−0.418	0.001	−0.398	0.001
Change in HADS-anxiety	−0.328	0.004	−0.269	0.031	−0.253	0.045
Change in HADS-depression	−0.420	<0.001	−0.397	0.001	−0.388	0.002

T2 − T0	*R*	*P*	*R*	*P*	*R* ^a^	*P* ^a^

Change in PSQI	−0.254	0.030	−0.099	0.442	−0.127	0.330
Change in PSQI-subjective sleep quality	−0.377	0.001	−0.272	0.031	−0.311	0.014
Change in PSQI-sleep latency	−0.255	0.027	−0.205	0.105	−0.222	0.080
Change in PSQI-sleep duration	−0.147	0.209	0.020	0.874	0.001	0.991
Change in PSQI-sleep efficiency	−0.071	0.548	0.126	0.322	0.110	0.393
Change in PSQI-sleep disturbance	−0.336	0.003	−0.235	0.062	−0.228	0.072
Change in PSQI-use of sleep medication	0.027	0.822	0.040	0.754	0.012	0.927
Change in PSQI-daytime dysfunction	−0.256	0.029	−0.157	0.222	−0.159	0.222
Change in total fatigue	−0.611	<0.001	−0.403	0.001	−0.360	0.004
Change in HADS-anxiety	−0.274	0.018	−0.313	0.012	−0.297	0.018
Change in HADS-depression	−0.286	0.013	−0.299	0.016	−0.292	0.020

T0: baseline; T1: immediate postintervention; T2: 3-month postintervention.

^a^Partial correlation controlling the amount of other exercises.
